# Economic Burden of Obesity: A Systematic Literature Review

**DOI:** 10.3390/ijerph14040435

**Published:** 2017-04-19

**Authors:** Maximilian Tremmel, Ulf-G. Gerdtham, Peter M. Nilsson, Sanjib Saha

**Affiliations:** 1 Institute for Medical Informatics, Biometry and Epidemiology (IBE), LMU Munich, 81377 Bavaria, Germany; 2 Health Economics Unit, Department of Clinical Sciences, Lund University, 22381 Lund, Sweden; ulf.gerdtham@med.lu.se (U.-G.G.); sanjib.saha@med.lu.se (S.S.); 3 Centre for Primary Health Care Research, Faculty of Medicine, Lund University/Region Skåne, Skåne University Hospital, S-22241 Lund, Skåne, Sweden; 4 Department of Economics, Lund University, S-22363 Lund, Skåne, Sweden; 5 Department of Clinical Sciences, Lund University, Skåne University Hospital, S-20502 Malmö, Skåne, Sweden; peter.nilsson@med.lu.se

**Keywords:** obesity, cost of illness, obesity-related disease, burden of obesity

## Abstract

*Background*: The rising prevalence of obesity represents an important public health issue. An assessment of its costs may be useful in providing recommendations for policy and decision makers. This systematic review aimed to assess the economic burden of obesity and to identify, measure and describe the different obesity-related diseases included in the selected studies. *Methods*: A systematic literature search of studies in the English language was carried out in Medline (PubMed) and Web of Science databases to select cost-of-illness studies calculating the cost of obesity in a study population aged ≥18 years with obesity, as defined by a body mass index of ≥30 kg/m², for the whole selected country. The time frame for the analysis was January 2011 to September 2016. *Results*: The included twenty three studies reported a substantial economic burden of obesity in both developed and developing countries. There was considerable heterogeneity in methodological approaches, target populations, study time frames, and perspectives. This prevents an informative comparison between most of the studies. Specifically, there was great variety in the included obesity-related diseases and complications among the studies. *Conclusions*: There is an urgent need for public health measures to prevent obesity in order to save societal resources. Moreover, international consensus is required on standardized methods to calculate the cost of obesity to improve homogeneity and comparability. This aspect should also be considered when including obesity-related diseases.

## 1. Introduction

Obesity is a condition in which fat accumulates in the body to a point where it is a risk factor or marker for a number of chronic diseases including diabetes, cardiovascular diseases (CVDs) and cancer, and has adverse effects on overall health [1,2,3]. Body mass index (BMI), calculated as weight in kilogram (kg) divided by height in meters squared, is one of the most commonly used screening tools to measure and characterize obesity. A BMI of 25 to <30 kg/m² is defined as overweight and BMI ≥ 30 kg/m² is classified as obese [4,5].

Obesity constitutes an important threat to national and global public health in terms of prevalence, incidence and economic burden. In 2014, more than 2.1 billion people, nearly 30% of the global population, were overweight or obese and 5% of the deaths worldwide were attributable to obesity. If the incidence continues at this rate, almost half of the world’s adult population will be overweight or obese by 2030 [6].

Obesity also imposes a large economic burden on the individual, and on families and nations [7,8]. In 2014 the global economic impact of obesity was estimated to be US $2.0 trillion or 2.8% of the global gross domestic product (GDP) [6]. Besides excess health care expenditure, obesity also imposes costs in the form of lost productivity and foregone economic growth as a result of lost work days, lower productivity at work, mortality and permanent disability. It has been described in recent studies and reviews that there is a gradient between increasing BMI and costs attributable to obesity [9,10,11,12].

Cost of illness (COI) studies help policy makers understand the economic burden of a specific disease. Such COI studies identify different components of costs of specific diseases or disease-related complications in different sectors of the society, which may have been saved if the disease had not existed. They are conducted from different perspectives that determine the types of cost included in the analysis. These perspectives measure costs to the society, health care systems, participants and their families and third-party payers [13,14]. Furthermore, COI studies have a significant role in public health in formulating and prioritizing health care policies and allocating health care resources by estimating the amount of costs attributable to a disease [15].

Systematic literature reviews represent a systematic way to identify relevant studies, to summarize the results, to critically analyse the methods of the studies and, finally, to comment and recommend improvements for future research. Systematic reviews in the context of cost of obesity (COO) summarize the results of available studies in order to provide a high level of evidence on the cost burden due to obesity, which may help decision makers to develop policies to tackle the burden of obesity [16].

There have been a number of literature reviews on COO [17,18,19,20,21,22,23,24,25,26,27,28] including studies from before 2011. Since 2011, however, advanced methods such as microsimulation modelling [29,30,31] have been used and have led to new findings, requiring further, systematic exploration. Furthermore, some reviews have included studies that were specific to a single country or continent, e.g., the USA [18,26], Canada [24] or Europe [21,22,27], and have excluded studies from all over the world. Some reviews have included studies that accounted for direct costs [23,26,28], while others have included only indirect costs [25]. Direct costs include all direct medical and non-medical costs for diagnosis, treatment and transportation [32]. Indirect costs are the productivity loss cost due to morbidity and early mortality [33]. Moreover, some studies include costs for both overweight and obesity and do not separately differentiate the cost burden [21,26].

In addition, none of the reviews has systematically analysed the obesity-related co-morbidities included in the cost calculation. Since obesity itself is not only a disease but also a risk factor for other diseases, it is important to study which co-morbidities have been included in the different COO analyses. The attributable burden of obesity differs across studies. Attributable burden is determined by the co-morbidities included in a cost calculation. It would be interesting to examine how, in the included studies, these co-morbidites are adjusted for in the overall cost calculation. 

Two recently published systematic reviews have attempted to explore the problems associated with the methodological heterogeneity of studies [10] and performed a quality appraisal of the analysed studies [12]. Nevertheless, there is still a methodological heterogeneity within COO studies and a lack of systematic reviews examining the different obesity-related diseases included in these studies.

The objective of this study was to: (1) perform a systematic review to assess the economic burden of adult obesity; and (2) identify and describe different obesity-related diseases included in the selected studies.

## 2. Methodology

This systematic review has been performed in accordance with the Preferred Reporting Items for Systematic Reviews and Meta-Analyses guidelines [34]. Moreover, the Campbell and Cochrane Economics Methods Group guidelines have been followed including search criteria, data extraction, synthesis and critical analysis.

### 2.1. Search Strategy

A systematic search was performed to identify relevant articles published in databases from 1 January 2011 until 14 September 2016. The databases used were Medline and Web of Science. Additional publications were searched on Google Scholar from the reference lists of included studies and reviews by backward and forward snowball searches. The details of the searching strategy with key words and initial hits are provided in Appendix A to ensure reproducibility and transparency of the work.

### 2.2. Inclusion and Exclusion Criteria

We included studies that satisfied the following criteria: (1) obesity was defined as BMI ≥ 30 kg/m²; (2) the estimation was based on the entire country and a representative population; (3) the estimated COO could be either direct or indirect or both; (4) estimated costs were specific to obesity and not overweight; (5) the research was reported in English in a peer-reviewed journal; and (6) studies with any perspective (e.g., societal, health care or third-party payers) in cost estimations.

Studies were excluded if they were: (1) economic evaluations such as cost-effectiveness, cost-utility or cost-benefit analyses; (2) reviews, notes, commentaries, or editorials related to obesity; or (3) COO studies that included children aged <18 years and pregnant women; (4) Articles describing study protocol or study design were likewise excluded.

### 2.3. Selection and Data Extraction

**Figure 1:**
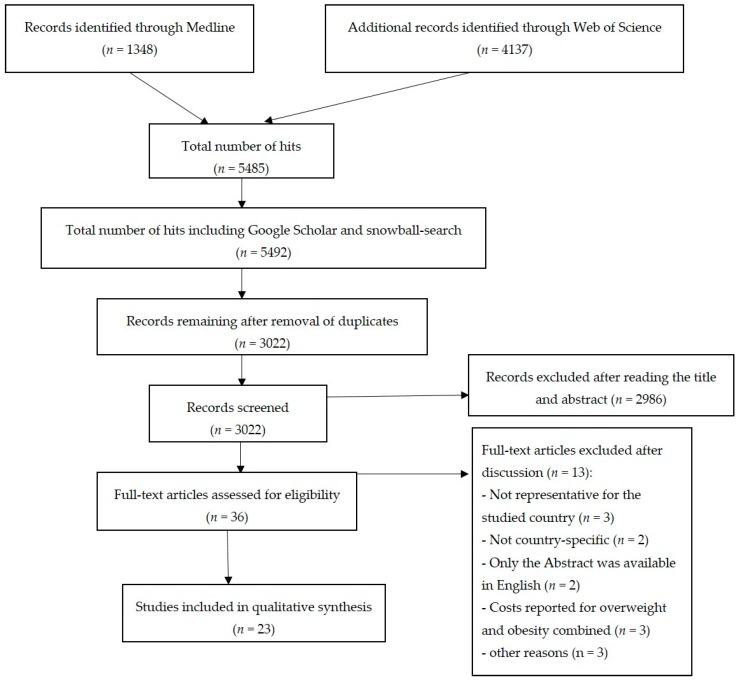
Flow chart depicting the process of the study selection for the systematic review.

Following each search in the above mentioned databases, the initial hits were exported into EndNote. After removing the duplicates, all titles and abstracts were screened to select the relevant studies based on the inclusion and exclusion criteria. The selection of the papers was done separately by two of the co-authors (Maximilian Tremmel and Sanjib Saha) who then checked the comparability of studies by reviewing a random sample of included and excluded studies after the initial screening. After removing studies that met the exclusion criteria during the initial screening, the full text of the remaining studies was assessed against the inclusion criteria and any differences were discussed and a consensus was reached. A flow chart of the study selection procedure is presented in Figure 1.

Data were extracted on two issues: (1) the results; and (2) the methodology used to derive the results. Other information was gathered as well, such as perspective, study time frame, sample size, target group, inclusion of cost items, and discount rate. Moreover, we also collected information on types of obesity-related co-morbidities included in the studies.

## 3. Results

We included twenty-three studies in this review [29,30,31,35,36,37,38,39,40,41,42,43,44,45,46,47,48,49,50,51,52,53,54]. Detailed characteristics of these studies are presented in Table 1. Eleven studies [29,30,38,39,40,44,45,46,50,53,54] used a top-down (population-based) approach and eleven studies applied a bottom-up (person-based) approach [31,35,36,37,41,42,43,47,48,51,52] to calculate the costs attributable to obesity. The top-down approach estimates economic costs by using aggregate data on mortality, morbidity, hospital admissions, general practice consultations, disease-related costs, and other health-related indicators along with population attributable fraction (PAF) or population attributable risk (PAR) to calculate attributable costs [55,56,57]. The measures of PAF and/or PAR were used in seven studies [38,40,44,45,46,50,53], while four studies did not mention the approach to estimating the costs [29,30,39,41]. One study [54] used population attributable prevalence (PAP), which takes into account that risk factors and their relative risks (RRs) can change over time.

The bottom-up approach calculates the resources used and productivity loss in individuals with the health problem in question, obesity in this case. The per-capita costs are then extrapolated to the whole population with the health problem, based on relevant epidemiological data [58]. The items that were included in the estimation of the patient-level data included drug medication in all twelve studies, but the other items, e.g., hospitalization costs, physician visit costs, inpatient and outpatient costs, varied across all studies. For example, whereas An [36] included out-of-pocket expenses, inpatient and outpatient costs, office-based medical provider services, emergency room services and medication, Effertz et al. [42] considered nursing costs, rehabilitation treatments, and financial compensations for job integrations, accidents, and medication.

There were 17 studies from developed countries [31,35,36,37,39,41,42,43,44,45,46,47,48,49,51,52,54] and six studies from developing countries [29,30,38,40,50,53] according to the World Economic Situation and Prospects (WESP) report. According to the WESP and Organization for Economic Co-operation and Development (OECD), there is no established convention for the designation of “developed” and “developing” countries, but in common practice, Japan, Canada, the USA and European countries, for example, are considered “developed” countries, while Mexico and Brazil are considered to be “developing” countries [59]. There were six studies from Germany [42,43,44,46,47,54], six from the USA [31,36,37,39,51,52], three from Brazil [30,38,40] and two from Canada [35,45].

In five studies [36,39,48,51,52] two-part models were used to calculate the health care expenses attributable to obesity. In two-part models, the probability of the medical expenditures is calculated first; thereafter it is multiplied by the amount of expenses conditional on the presence of these expenses. A microsimulation model was designed and applied by Rtveladze et al. for Brazil [30] and Mexico [29]. Both these studies employed the two-stage modelling process developed by the UK Foresight working group [60] and results were simulated for three hypothetical scenarios (no BMI reduction, a 1% reduction, or a 5% reduction in BMI across the population). The model predicted the costs for Mexico to rise from US $806 million (2010) to US $1.7 billion in 2050. For Brazil, the costs were estimated to increase from US $5.8 billion (2010) to US $10.1 billion (2050). Another microsimulation model (Markov-based microsimulation) was developed by Su et al. [31], which predicted the 5-year and 10-year total economic burden per capita attributable to obesity at US $33,900 and US $70,200 (2013), respectively.

Studies also varied in terms of inclusion of direct costs and indirect costs, i.e., in terms of perspective of analysis (Table 1). Direct medical costs include costs for the treatment and management of the diseases, e.g., inpatient or outpatient care. Direct non-medical costs include, e.g., transportation costs to health care providers. Indirect costs include early mortality costs and morbidity costs due to sickness absence and informal care costs [13]. In six studies [42,44,45,46,50,53], both direct and indirect costs were included and therefore a societal perspective was used. In twelve studies [29,30,35,36,38,39,40,41,43,48,51,52] only direct costs were calculated and therefore a health care perspective was used. However, one of these studies [43] described this method as a societal perspective rather than a health care perspective. 

Indirect costs only were calculated in two studies [47,49]. In a study from the USA [52], direct costs were estimated from a third-party payer perspective and in another study from Germany [42] both direct and indirect costs were estimated from a third-party payer perspective. The third-party payer perspective includes insurance companies, governmental agencies, and employers. The Medicaid perspective, a government programme financed by federal, state and local funds for persons of all ages within certain income limits, was used in the U.S. study while in the German study, the perspective of the “Techniker Krankenkasse” insurance company was used. The informal costs and informal caregiver costs were included in only two studies [43,53].

We found a substantial variation in the items that were included while estimating the direct cost (Table 1). For example, in one study from Brazil, by Bahia et al. [38], only inpatient and outpatient costs were included for the estimation of the direct costs, while in another Brazilian study, by de Oliveira et al. [40], costs for bariatric surgery, medication, orthotics, prosthetics, medical consultation and diagnostic procedures were additionally included. There was also variation in the calculation of indirect costs. Out of nine studies, in eight studies [42,44,45,46,47,49,50,53] researchers used the human capital approach (HCA) to calculate the indirect costs. Neovius et al. used the friction cost approach (FCA) as well as the HCA to estimate the indirect COO for Swedish men [49]. The HCA measures lost production, in terms of lost earnings of a patient. For mortality or permanent disability costs, the HCA multiplies the earnings lost at each age by the probability of living to that age [57]. In the FCA, only the production losses during the time it takes to replace a worker [57] are measured. Andreyeva et al. used average earnings to measure indirect costs [37].

We further gathered information on the obesity-related diseases included in each of the studies listed in Table 2. In 14 studies, researchers mentioned obesity-related diseases in the cost calculation [29,30,31,35,38,40,44,45,46,47,50,52,53,54]. The costs of diabetes were included in all of these 14 studies, three of which [35,51,53] included both Type 1 and Type 2 diabetes. Additionally, all of the studies, except one [35], considered CVDs. Therefore, diabetes and CVDs were the most commonly considered comorbidities of obesity in the selected studies. In addition to diabetes and CVDs regarded as comorbidities of obesity, both hypertension [29,30,31,35,38,40,44,46,47,50,53,54] and cancer [29,30,31,37,40,44,45,46,50,52,53,54] were included in twelve studies. However, these studies differ with regard to the type of cancer included in the cost analysis.

For example, Konnopka et al. [44] included neoplasms of the oesophagus, stomach, colon, liver, gallbladder, pancreas, postmenopausal breast, cervix uteri, ovary, prostate, and kidney, non-Hodgkin’s lymphoma, multiple myeloma, and leukaemia, while Kang et al. [53] included only colon cancer among the cancers. Furthermore, musculoskeletal disorders were considered in nine [29,30,31,38,40,45,50,53,54], respiratory disorders in six [31,38,40,45,50,52] and digestive diseases in five studies [31,44,45,46,50]. Four studies [31,35,47,50] have also included mental disorders such as depression. All of the abovementioned diseases were included only in the studies by Pitayatienanan et al. [50] and Su et al. [31].

Two studies estimated the obesity burden in Brazil from a health care perspective. Bahia et al. [38] calculated the costs over 3 years from 2008 to 2010 to be US $1.1 billion per year and de Oliveira et al. [40] gave the burden of obesity in 2010 as US $269.6 million. Both studies used the PAF and a top-down approach. Bahia et al. [38] collected data from the national health database from 2008 to 2010 and the costs reflected the average costs for 3 years. De Oliveira et al. [40] used Ministry of Health Data to estimate the annual health care costs.

Konnopka et al. [44] used the concept of attributable fractions based on German prevalence data and relative risks from US studies as well as statistics from the German Federal Statistics Office and the German Retirement Insurance Office. These results were updated by Lehnert et al. [46] 6 years later using the same method to calculate the cost burden. The total annual societal (direct and indirect) costs due to obesity increased from €9.8 million in 2002 to €12.2 million in 2008. Another study from Germany [42], using a different method based on claims data from a German health insurance company, estimated the total costs for third-party payers to be €63.0 billion per year. Konig et al. [43] estimated the average 3-month individual health care costs (also including informal care) in Germany to be €1244 (2008) using questionnaire data from an 8-year follow-up contact of a large population-based prospective cohort study titled “Epidemiologische Studie zu Chancen der Verhütung, Früherkennung und optimierten Therapie chronischer Erkrankungen in der älteren Bevölkerung” (the ESTHER study). Yet another German study [54] estimated the total national health care costs at €5.1 billion, using the OBCOST tool to estimate incidence, prevalence and mortality (IPM) to calculate the COO.

For Canada, the annual societal costs were estimated to be CAD $1.0 billion, according to Krueger et al. [45] using data from the 2012 Canadian Community Health Survey. Kang et al. [53] included 1,910,194 Korean individuals in their study to calculate the annual societal costs, which in 2005 amounted to US $1786 billion. Annual societal costs were also estimated in a study in Thailand [50] and costs attributable to obesity were US $725.3 million in 2009. For Sweden, Neovius et al. [49] estimated that the total lifetime productivity loss due to obesity was €95,400 per man in 2003. This study was based on a 38-year follow-up of 45,920 Swedish men who were performing mandatory military conscription tests at age 18.7 ± 0.5 years.

Direct per-capita costs of obesity were reported in seven studies [31,35,36,37,43,48,52] and indirect per-capita costs were calculated in one study in Sweden [49]. When comparing the results of two studies in the USA [36,39] estimating annual direct costs per capita, the costs increased from US $2741 in 2005 to US $6899 in 2011. Both these studies used data from the Medical Expenditure Panel Survey. Alter et al. [35] estimated the direct per-capita costs attributable to obesity over a time frame of 11.5 years to be CAD $8294.67 (2006) while the direct per-capita costs over a lifetime (>65 years) amounted to US $171,482 (2010) in the USA [52]. Total per-capita costs in the USA were predicted, using a Markov-based microsimulation, to be US $33,900 and US $70,200 (2013) over a time frame of 5 and 10 years, respectively [31].

## 4. Discussion

In this paper, we have performed a systematic literature review of recent cost of obesity (COO) studies. We have found that there is still a large heterogeneity across the available COO literature. Although there is a substantial international literature on COO, we have found that a review and synthesis of the results based on homogeneous methods and costing approaches is hindered by a wide range of sources, as well as methodological approaches, perspectives, target groups and included diseases, used to estimate the prevalence of obesity.

A key issue of COI studies is the PAF applied to calculate the fraction of costs attributable to obesity. There are no agreed recommendations or guidelines on what fraction of the co-morbidities can be attributed to obesity and what fraction can be attributed to the co-morbidities themselves. Since obesity is a complex disease condition with much different co-morbidity, what fraction of the co-morbidities is attributed to obesity has much influence on the cost calculation. The PAF is calculated by using the RRs for co-morbidities related to obesity. In the literature review, we found different methods for calculation of RRs and, subsequently, PAF. For example, Lette et al. [54] applied age- and gender-specific RRs and used obesity-related co-morbidities from the Comparative Quantification of Health Risks [61]. Bahia et al. [38] selected co-morbidities based on two conditions: firstly, those RRs are ≥1.20 for diseases and secondly, that RRs are ≥1.10 but <1.20 for diseases that are a substantial problem for public health due to high prevalence rate. The authors calculated the RRs by performing meta-analyses. The different methods for calculation of PAF can lead to an over—or an underestimation of costs attributable to obesity and can therefore make it difficult for comparison between studies.

Our literature review included studies that are based on different approaches for calculating the disease burden of obesity (Table 1). Each approach has advantages and disadvantages. The top-down approach is simple, transparent, and cheaper and faster than the bottom-up approach. A disadvantage of the top-down approach is that all possible confounding variables need to be adjusted for when estimating the PAF. For a complex disease such as obesity, this approach may underestimate or overestimate the costs derived from co-morbidities. The bottom-up approach, on the other hand, calculates the mean per-person costs, which are then extrapolated to the whole population. In this case, the patient sample size needs to be unbiased and representative of the national population. This might require extensive resources and may not be always practical (e.g., for estimating the future cost) [62]. On the other hand, this approach is more comprehensive and valid, and enables detection of the variability related to differences in important demographic characteristics between patients [58]. Microsimulation models can predict the future cost and can incorporate data from other countries, if data are missing in a specific case or if data from another country are known to be valid and sufficiently reliable to be incorporated. A disadvantage of microsimulations is that a number of assumptions are made that may or may not be valid; these assumptions have to be checked using sensitivity analysis to evaluate how sensitive indicators can react to changes in input parameters. This process makes the model complex and sometimes makes it difficult to understand [63].

The study by Lehnert et al. [46] aimed to update the study by Konnopka et al. [44] and used the same method, perspective and target group in Germany. Therefore, these studies provide a good picture of the increase in the societal COO in Germany, from €9.8 million in 2002 to €12.2 million in 2008. Researchers argued that the main driver behind the cost increases was the rise in the prevalence of overweight and obesity in Germany between 2002 and 2008. This series of studies from Germany, using the same methods to measure the COO, may provide a valid statement about the development of COO between these two time points and gives a good example of how COO studies can be conducted in a structured and valid way. Nevertheless, the costs estimated in these two studies differ crucially from those reported by Krueger et al. [45] who used a similar approach to estimate the annual COO in Canada. Although the population of Canada is less than half of the population of Germany, these authors estimated the annual COO at CAD $1.0 billion. This variation in estimated costs can be explained by the approaches to calculating the risk factor exposure of obesity. The two studies from Germany used relative risks (RR) data from studies conducted in the USA to calculate the PAF. Even though estimates of RR were adjusted for important confounders such as gender, age, race and smoking status in both studies, transfer of costs to the German population causes uncertainty. By contrast, the study from Canada used RR data from a previously conducted literature review on studies of the general population of Western countries. Whereas Konnopka et al. [44] used German prevalence data and RRs from the US studies, Krueger et al. [45] used self-reported data from the Canadian Community Health Survey to calculate the risk factor exposure. Moreover, the two studies included different diseases in the cost calculation. Krueger et al. [45] excluded hypertension while Konnopka et al. [44] excluded respiratory and musculoskeletal disorders in the costing approach, which may explain some of the variation in estimated costs.

We included three studies from Brazil which calculated direct COO. Bahia et al. [38] collected data of the national health database from 2008 to 2010 and their estimated cost of US $1.1 billion reflects the average of 3 years. De Oliveira et al. [40] also used a top-down approach with Ministry of Health data to estimate the annual health care costs, which amounted to US $269.6 million. Rtveladze et al. [30] used a microsimulation model (Monte Carlo simulation), which requires county-specific disease incidence data, to predict health care costs from 2010 to 2050. Their results are limited by the lack of country-specific incidence and, e.g., cancer mortality data, as they used data from the USA, which has led to an overestimation of costs because Brazilian per capita health care spending is nearly eight times lower compared with the USA. When comparing these three study results, several limitations have to be pointed out: e.g., Bahia et al. [38] used RR data from countries other than Brazil since no data were available based on Brazilian cohorts. In addition, obesity prevalence rates were obtained from self-reported weight and height, which method may lead to either overestimation or underestimation of costs attributable to obesity, when either too many or too few people are categorized as obese, based on self-reported weight and height. On the other hand, de Oliveira et al. [40] used the PAR of obesity to calculate the costs for morbid obesity, which can lead to an underestimation of costs; also, they obtained RR data from cohort studies and meta-analyses published in international journals. Consequently, the different data sources used to estimate the RR relevant for the cost calculation need to be considered. When comparing these costs with costs in developed countries, it should be borne in mind that the Brazilian public health system has a large unmet demand for bariatric surgery, and consequently, that there may be an underestimation of COO in Brazil due to unmet needs [64].

Another characteristic of studies included in this review was the limited time frame of the analyses. In only six studies [31,35,38,48,49,52] was the time frame of the analyses longer than 1 year. Su et al. [31] reported per-capita costs in the USA over a time frame of 5 and 10 years. Alter et al. [35] investigated a time frame of 11.5 years to estimate the cumulative per-capita costs. Additionally, a propensity score matching method based on important confounders such as age, gender, socioeconomic status, smoking, physical activity, psychosocial stress and comorbidity, and a sensitivity analysis were performed, but the results did not change. Nevertheless, these results are limited by the exclusion of patients aged 65 and older, which may imply an underestimation of the costs and hinder a useful comparison with, e.g., the study by Yang et al. [52], who calculated lifetime costs from 65 years onwards.

Some studies failed to incorporate a discount rate (Table 1) [29,30,31,35,36,37,38,39,40,41,43,45,47,48,51,52,54]. Discounting allows calculation of the present value of payments that will be made in the future and should be applied when the duration of the analysis is longer than 1 year, otherwise the calculated costs might overestimate the true costs. Effertz et al. [42] incorporated discounting in a 1-year time frame of analysis, whereas for example Alter et al. [35] did not apply any discounting over a time frame of 11.5 years. Furthermore, the discount rates also vary among studies. Effertz et al. [42] used a discount rate of 2% while Kang et al. [53] discounted the costs at a rate of 6%. Hence, the costs reported by Effertz et al. [42] might overestimate the true costs, while the costs calculated by Kang et al. [53] might underestimate them. There is no agreement on the discount rate to be used in the scientific literature, although the World Health Organization (WHO) has recommended using a 3% discount rate [65].

Moreover, it should be pointed out that only four studies [31,35,47,50] include costs for mental disorders as a relevant obesity-related disease. According to Vigo et al. [66], the burden of mental disorders still seems to be underestimated even though e.g., depression as a mental disorder is on the rise globally, according to the WHO [67]. A recent systematic review [68] investigated the relationship between obesity and depression among adult men and women. The results indicate that there is a bidirectional relationship between obesity and depression. Consequently, excluding depression and other mental disorders from the obesity-related diseases may lead to an underestimation of costs. For example, the societal costs of depression in Germany were estimated at €15.6 billion per year [69].

The International Agency for Research into Cancer (IARC) [70] and the World Cancer Research Fund (WCRF) [71] report that common cancers in obese people are endometrial, oesophageal, colorectal, postmenopausal breast, prostate and renal cancer and adenocarcinoma. Less common malignancies associated with obesity are malignant melanoma, thyroid cancers [72], leukaemia, non-Hodgkin’s lymphoma, and multiple myeloma [73]. However, there was a crucial heterogeneity between the studies that included different types of cancer. Kang et al. [53] only included colon cancer as an obesity-related disease, while Konnopka et al. [44] and Lehnert et al. [46] included stomach, kidney, liver, gallbladder, cervix, ovary cancers and non-Hodgkin’s lymphoma, multiple myeloma, and leukaemia in addition to the common cancers in obese people mentioned by the IARC and WCRF. Su et al. [31] included 16 different types of obesity-related cancers in their study. The reported costs due to cancers need to be interpreted with the knowledge that different types of cancer were included in the different studies, which may have led to over- or underestimation of costs. Due to the fact that cancers create a big cost burden for society [74], there is a need for standardization when including cancers in the obesity-related costs. Within the twelve studies that have mentioned the included obesity-related diseases, one study, by Su et al. [31], included obesity-related liver diseases, such as non-alcoholic fatty liver disease (NAFLD), liver fibrosis and cirrhosis of the liver, which are also associated with obesity [75,76]. For example, NAFLD, a very common chronic liver disease worldwide, is on the rise following the trend of increasing prevalence of obesity, and is the second most common indication for liver transplantation, and an important cause of hepatocellular carcinoma [77]. Also, hepatic steatosis is known to be an associated comorbidity of obesity [78]. Consequently, we recommend considering liver diseases when costs of obesity and related diseases are calculated.

We found three studies, from the USA [37], Germany [47] and Sweden [49], in which only indirect costs due to obesity were calculated. While Andreyeva et al. [37] used overall average earnings to calculate the costs, Lehnert et al. [47] and Neovius et al. [49] used the HCA. Therefore, it has to be noted that using overall average earnings may overestimate average earnings for obese workers, especially women, in light of evidence that obesity is associated with low socioeconomic status [79]. Neovius et al. [49] state that using an FCA, compared with the HCA, reduced the estimated productivity losses by about 80%. Therefore, it may be beneficial to calculate indirect costs both using HCA and FCA approach.

Summarizing these results, we can state that obesity is responsible for a large fraction of costs, not only to the health care system but also to society at large. As we stated previously, almost half of the world’s adult population will be overweight or obese by 2030 if the prevalence continues on the current trend [6] and consequently also the costs attributable to obesity will increase. A useful example for rapidly rising costs attributable to obesity from these selected studies are the two mentioned studies from Germany [44,46]. The results of the two papers together show that total societal costs in Germany due to obesity have increased from €9.8 million to €12.2 million between 2002 and 2008. Therefore, public health interventions should focus on the prevention of obesity as soon as possible, ideally at a young age. A possible option would be to focus on work site health promotion (WHP) to increase physical activity and healthy lifestyles at the workplace, especially as obesity has been found to be associated with absenteeism, disability pension and overall work impairment [80]. Higher physical activity at work may not only lead to a reduction in BMI and obesity, but also increase the health status of the employees. This may in turn further reduce indirect costs due to absenteeism and disability pension.

Furthermore, the definition of the various perspectives used in the studies should be discussed, since the term “societal” as a perspective was used variously in different studies. The societal perspective should include all costs (direct and indirect) except transfer payments (a shift of resources such as social security benefits or Medicare or Medicaid payments) [57]. For example, Lehnert et al. [47] and Neovius et al. [49] who only calculated indirect costs of obesity described theirs as a societal perspective. Konig et al. [43] only estimated direct costs, yet also used the term “societal” to describe their perspective.

A limitation of this review is that we only used Medline, Web of Science and Google Scholar to search for studies, which may have limited the number of potentially eligible studies. In addition, we only examined articles published in English. Furthermore, the absence of international standardized methods and considerable heterogeneity between the study designs of these COO studies hinders the completion of a comprehensive review. Another limitation of this study is that we did not aim to perform a quality appraisal of the selected studies, also due to the fact that there are no validated guidelines to perform a quality check for COI studies. Furthermore, we considered obesity to be a fixed condition even though it has been discussed in the recent literature that obesity may be a transient state, e.g., depending on age cohorts or period effects [81].

## 5. Conclusions

The studies under review show that obesity is responsible for a large fraction of costs, both for health care systems and for society. Heterogeneity is a major limitation among the COI literature in general and the COO literature in particular, which hinders a conclusive comparison of the different studies. We recommend that obesity-related diseases and complications should be included more consistently. We also recommend that additional obesity-related diseases be considered in further COO studies, such as liver and mental diseases which have mostly been neglected so far.

## Figures and Tables

**Table 1:** Characteristics of the included studies.

Author, Publication Year, Country	Objective	Perspective	Time Frame	Sample Size	Target Group	Cost as Reported (Costing Year)	Direct Costs Included Items	Method	Indirect Costs Included Items	Method	Discount Rate
Alter et al., 2012, Canada [35]	To estimate long-term health care expenditures	Health care	11.5 years	9398	<65 years, BMI ≥ 18.5 and without pre-existing heart disease	Cumulative per-capita costs over whole time frame: CAD $8294.67 (2006)	Hospitalization costs, visits to the GP, medication, cardiac procedural costs	Prevalence-based, bottom-up approach, retrospective	Not included	Not relevant	N.M.
An, 2015, USA [36]	To estimate annual health care expenses by modelling	Health care	1 year	125,955	≥18 years	Annual per-capita costs: US $6899 (2011)	Out-of-pocket expenses, inpatient and outpatient costs, office-based medical provider services, emergency room services, medication	2 PM; Prevalence-based, bottom-up approach, retrospective	Not included	Not relevant	N.M.
Andreyeva et al., 2014, USA [37]	To estimate annual productivity loss	Societal *	1 year	14,975	Employed American adults	US $8.65 billion (2012)	Not included	Prevalence-based, bottom-up approach, retrospective	Loss of productivity due to work absenteeism	Overall average earnings	N.M.
Bahia et al., 2012, Brazil [38]	To estimate health care costs	Health care	3 years	54,339	Brazilians ≥18 years	US $1.1 billion (2010)	Inpatient and outpatient costs	Prevalence-based, top-down approach, retrospective	Not included	Not relevant	N.M.
Cawley & Meyer-hoefer, 2012, USA [39]	To estimate annual direct health care costs	Health care	1 year	23,689	20–64 years	Annual per-capita costs: US $2741 (2005)	Inpatient and outpatient costs, medication, dental, vision, home health care services and medical equipment	2 PM; prevalence-based, top-down approach, retrospective	Not included	Not relevant	N.M.
De Oliveira et al., 2015, Brazil [40]	To estimate annual direct health care costs	Health care	1 year	188,461	All Brazilians with access to the public health system	Total costs: US $269.6 million and 64.2 million for morbid obesity (2011)	Inpatient and outpatient costs, bariatric surgery, medications, orthotics, prosthetics, medical consultations and diagnostic procedures	Prevalence-based, top-down approach, retrospective	Not included	Not relevant	N.M.
Doherty et al., 2012, Republic of Ireland [41]	To estimate health care costs	Health care	1 year	10,184	≥18 years	Total costs: 31.5 million (primary & secondary health care) (-)	Visits to the GP, inpatient costs, day case (inpatient)	Bottom-up approach, retrospective	Not included	Not relevant	N.M.
Effertz et al., 2015, Germany [42]	To estimate annual societal costs	Third-party payer	1 year	146,000	Insured population in Germany	Total costs: €63.04 billion; Direct costs: €29.39 billion; Indirect costs: €33.65 billion (-)	Nursing costs, rehabilitation treatments, financial compensations for job integrations, accidents, medication	Prevalence-based, bottom-up approach, retrospective	Sickness absence, nursing care, early retirement pension, pension for widows and orphans, rehabilitation, unemployment, premature mortality	HCA	2%
Kang et al., 2011, Korea [53]	To estimate annual societal costs	Societal	1 year	1,910,194	Population aged ≥ 20 years	Total costs: US $1786 billion Direct costs: US $1080 billion Indirect costs: US $705.8 million (2005)	Inpatient and outpatient costs and medication	Incidence-based, top-down approach, retrospective	Loss of productivity due to premature mortality and sickness absence; time costs, traffic costs and nursing fees	HCA	6%
Konnopka et al., 2011, Germany [44]	To estimate annual societal costs	Societal	1 year	Entire population	Entire adult population	Total costs: €9.873 million Direct costs: €4.854 million Indirect costs: €5.019 million (2002)	Inpatient and outpatient costs, rehabilitation, administration and research	Prevalence-based, top-down approach, retrospective	Loss of productivity due to sickness absence, early retirement and premature mortality	HCA	5%
Konig et al., 2015, Germany [43]	To estimate societal costs	Societal	3 months	3108	Population aged 58–82	Direct per-capita costs: €1244 (2008)	Inpatient and outpatient costs, medication, dental prostheses, professional community nursing home care and informal care	Population-based, bottom-up approach, retrospective	Not included	Not relevant	N.M.
Krueger et al., 2015, Canada [45]	To predict annual societal costs by simulation modelling	Societal	1 year	-	17–100 years	CAD $1.0 billion (2013)	Hospital care, physician services, medication, health research and other health care expenditures	Prevalence-based, top-down approach, retrospective	Loss of productivity due to short-term disability, long-term disability and premature mortality	HCA	N.M.
Lehnert et al., 2015, Germany [46]	To estimate annual societal costs	Societal	1 year	Entire population	Entire adult population	Total costs: €12.2 million Direct costs: €6.05 million Indirect costs: €6.19 million (2008)	Inpatient and outpatient costs, rehabilitation, health protection, ambulance, administration, research, investments and education	Prevalence-based, top-down approach, retrospective	Loss of productivity due to sickness absence, early retirement and premature mortality	HCA	5%
Lehnert et al., 2014, Germany [47]	To estimate annual productivity loss	Societal *	1 year	7990	18–65 years and employed	Annual per capita costs: €772.0 (2009)	Not included	Bottom-up approach, retrospective	Loss of productivity in paid work due to absenteeism	HCA	N.M.
Lette et al., 2016, Germany, The Netherlands, Czech Republic [54]	To estimate annual health care costs	Health care	1 year	Entire population	Population aged ≥ 20 years	Annual direct costs: DE: €5.1 billion; NL: €528.3 million; CZ: €108.3 million (-)	Not mentioned	Prevalence-based, top-down approach, retrospective	Not included	Not relevant	N.M.
Mora et al., 2015, Spain [48]	To estimate health care costs by modelling	Health care	7 years	452,108	Entire adult population	Annual per-capita costs: US $1382.42 Increase in annual per-capita costs: US $381.17 (2010)	Visits to the GP, specialist and emergency care, hospitalization, laboratory, radiology and other diagnostic tests and medication	2PM; Prevalence-based, bottom-up approach, prospective	Not included	Not relevant	N.M.
Neovius et al., 2012, Sweden [49]	To estimate lifetime productivity losses	Societal *	Lifetime (38 years)	45,920	19–65 years	Total lifetime productivity loss: €95,400 (2003)	Not included	Not relevant	Lifetime loss of productivity; sickness absence; disability pension and premature mortality	HCA (FCA)	3%
Pitayatienanan et al., 2014, Thailand [50]	To estimate annual societal costs	Societal *	1 year	N.M.	Entire adult population	Total costs: US $725.3 million Direct costs: US $333.6 million Indirect costs: US $391.8 million (2009)	Inpatient and outpatient costs	Prevalence-based, top-down approach, retrospective	Loss of productivity due to premature mortality and hospital-related absenteeism	HCA	3%
Rtveladze et al., 2014, Mexico [29]	To predict health care costs by microsimulation	Health care	1 year	Mexican adults	Entire adult population	Health care US $806 million (2010)	Total costs for health care and disease-related costs	Incidence-based, top-down approach, prospective	Not included	Not relevant	N.M.
Rtveladze et al., 2013, Brazil [30]	To predict health care costs by microsimulation	Health care	1 year	Brazilian adults	≥20 years	US $5.81 billion (2010)	Inpatient costs, medication, consultation, management of complications	Incidence- based, top-down approach, prospective	Not included	Not relevant	N.M.
Su et al., 2015, USA [31]	To predict societal costs by microsimulation	Societal	5 years	5221	20–85 years	Total per-capita costs: US $33,900 Direct per-capita costs: US $20,200 (2013)	N.M.	Bottom-up approach, prospective	Loss of productivity due to absenteeism and disability	N.M.	N.M.
Wang et al., 2015, USA [51]	To predict health care costs by modelling	Health care	1 year	117,948	All taxpayers and employers	US $69 billion for severe obesity (2014)	Bariatric surgery, nutrition consultation, weight loss programme, medication	2 PM; prevalence-based, bottom-up approach	Not included	Not relevant	N.M.
Yang & Zhang, 2014, USA [52]	To predict the societal costs by model simulation	Third-party payer	Lifetime (from 65 years on)	28,906	Entire adult population aged ≥ 65	Total lifespan per-capita costs: US $171,482 (2012)	Inpatient and outpatient costs, physician services, LTC, medication	2 PM; Incidence-based, bottom-up approach, prospective	Not included	Not relevant	N.M.

Abbreviations: 2 PM = two-part model; CZ = Czech Republic; DE = Germany; FCA = friction cost approach; GP = general practitioner; HCA = human capital approach; LTC = long-term care; NL = The Netherlands; N.M. = not mentioned; (-) = costing year was not mentioned; * including loss of productivity only.

**Table 2:** Obesity-related diseases included in the studies.

Author, Year, Country	Diabetes	CVDs	Hyper-Tension	Cancer	Respiratory Disorders	Musculo-Skeletal Disorders	Mental Dis-Orders	Digestive Diseases	Other
Alter et al., 2012, Canada [35]	√		√				√		
Bahia et al., 2012, Brazil [38]	√	√	√	√	√	√			
de Oliveira et al., 2015, Brazil [40]	√	√	√	√	√	√			
Kang et al., 2011, Korea [53]	√	√	√	√		√			√
Konnopka et al., 2011, Germany [44]	√	√	√	√				√	√
Krueger et al., 2015, Canada [45]	√	√		√	√	√		√	√
Lehnert et al., 2014, Germany [47]	√	√	√				√		√
Lehnert et al., 2015, German (UPDATE) [46]	√	√	√	√				√	√
Lette et al., 2016, DE, NL, CZ [54]	√	√	√	√		√			
Pitayatienanan et al., 2014, Thailand [50]	√	√	√	√	√	√	√	√	√
Rtveladze et al., 2014, Mexico [29]	√	√	√	√		√			
Rtveladze et al., 2013, Brazil [30]	√	√	√	√		√			
Su et al., 2015, USA [31]	√	√	√	√	√	√	√	√	√
Yang & Zhang, 2014, USA [52]	√	√		√	√				

Abbreviations: CVDs = cardiovascular diseases; CZ = Czech Republic; DE = Germany; NL = The Netherlands.

## Appendix A: Details of the Search Strategy with Keywords and Initial Hits.

MeSH-Terms used in MedLine (initial hits: 1348):

((((((((((((((cost [MeSH Terms]) OR absenteeism [MeSH Terms]) OR presenteeism [MeSH Terms]) OR productivity [MeSH Terms]) OR sick leave [MeSH Terms]) OR health cost, employer [MeSH Terms]) OR compensations, workers [MeSH Terms]) OR disability leaves [MeSH Terms]) OR premature mortality [MeSH Terms]) AND (”2011/01/01”[PDat]: “2016/09/13” [PDat]) AND Humans [Mesh] AND English [lang])) OR ((((((((((((costs and cost analysis [MeSH Terms])) OR economics [MeSH Terms]) OR cost benefit analysis [MeSH Terms]) OR cost allocation [MeSH Terms]) OR cost of illness [MeSH Terms]) OR cost control [MeSH Terms]) OR health care costs [MeSH Terms]) OR direct service costs [MeSH Terms]) OR hospital costs [MeSH Terms]) OR employer health costs [MeSH Terms]) OR drug costs [MeSH Terms])) AND (“2011/01/01” [PDat]: “2016/09/13” [PDat]) AND Humans [Mesh] AND English [lang])) AND (((((obesity, morbid [MeSH Terms]) OR ((((anti-obesity agents [MeSH Terms]) OR obesity, abdominal [MeSH Terms]) OR obesity [MeSH Terms]) OR overweight [MeSH Terms])) OR Abdominal obesity metabolic syndrome [MeSH Terms]) OR Anti-Obesity Agents [MeSH Terms]));

Terms used for Web of Science search (initial hits: 4137):

Absenteeism, presenteeism, productivity, sick leave, health cost, workers’ compensations, disability leaves, premature mortality, costs and cost analysis, economics, cost benefit analysis, cost allocation, cost of illness, cost control, health care costs, direct service costs, hospital costs, employer health costs, anti-obesity agents, abdominal obesity, obesity, overweight, abdominal obesity, metabolic syndrome, Anti-Obesity Agents;

Initial hits for both Medline and Web of Science: 5485
